# Metabolomic markers of fertility in bull seminal plasma

**DOI:** 10.1371/journal.pone.0195279

**Published:** 2018-04-10

**Authors:** Ana Luiza Cazaux Velho, Erika Menezes, Thu Dinh, Abdullah Kaya, Einko Topper, Arlindo Alencar Moura, Erdogan Memili

**Affiliations:** 1 Department of Animal and Dairy Sciences, Mississippi State University, Mississippi State, MS, United States of America; 2 Department of Animal Sciences, Federal University of Ceara, Fortaleza, Ceara, Brazil; 3 Alta Genetic Inc., Watertown, WI, United States of America; 4 Department of Reproduction and Artificial Insemination, Selcuk University, Konya, Turkey; Faculty of Animal Sciences and Food Engineering, University of São Paulo, BRAZIL

## Abstract

Metabolites play essential roles in biological systems, but detailed identities and significance of the seminal plasma metabolome related to bull fertility are still unknown. The objectives of this study were to determine the comprehensive metabolome of seminal plasma from Holstein bulls and to ascertain the potential of metabolites as biomarkers of bull fertility. The seminal plasma metabolome from 16 Holstein bulls with two fertility rates were determined by gas chromatography-mass spectrometry (GC-MS). Multivariate and univariate analyses of the data were performed, and the pathways associated with the seminal plasma metabolome were identified using bioinformatics approaches. Sixty-three metabolites were identified in the seminal plasma of all bulls. Fructose was the most abundant metabolite in the seminal fluid, followed for citric acid, lactic acid, urea and phosphoric acid. Androstenedione, 4-ketoglucose, D-xylofuranose, 2-oxoglutaric acid and erythronic acid represented the least predominant metabolites. Partial-Least Squares Discriminant Analysis (PLSDA) revealed a distinct separation between high and low fertility bulls. The metabolites with the greatest Variable Importance in Projection score (VIP > 2) were 2-oxoglutaric acid and fructose. Heat-map analysis, based on VIP score, and univariate analysis indicated that 2-oxoglutaric acid was less (*P* = 0.02); whereas fructose was greater (*P* = 0.02) in high fertility than in low fertility bulls. The current study is the first to describe the metabolome of bull seminal plasma using GC-MS and presented metabolites such as 2-oxoglutaric acid and fructose as potential biomarkers of bull fertility.

## Introduction

Male fertility relates to the capacity of an animal to produce spermatozoa with the ability to fertilize the oocyte, resulting in a living offspring. Fertility is affected by several factors, including management, nutrition, disease, stress, age, and genetics [1]. A decline in bull fertility affects the conception rate of herds, resulting in decreased production and profit. Therefore, the ability to predict bull fertility in advance offers enormous benefits for the economic success of livestock enterprise by improving pregnancy rates [2]. The “*omics*” approaches, such as genomics, transcriptomics and proteomics, have been used to ascertain molecular determinants of bull fertility. As a result, studies show that molecular compounds found in sperm and seminal plasma are significantly associated with bull fertility [3–6].

Seminal plasma is a complex mixture of secretions from testis, epididymis and accessory sex glands. Given the molecular contributions of the seminal plasma in sperm physiology, metabolites may affect downstream and complementary changes in gene and protein expressions and may be the underlying the key regulators of bull fertility [7]. Metabolites present in seminal plasma play several roles related to sperm function, such as energy production, motility, protection, pH control and regulation of metabolic activity [8].

Metabolomics is an emerging technique and has shown promise in identifying potential male fertility and infertility biomarkers [8–11]. Metabolomics represents the downstream of systems biology and has drawn significant interest for studying and understand fundamental biological processes related to reproduction [10, 12] since it allows the identification and quantification of small molecules, such as amino acids, peptides, fatty acid, and carbohydrates in secretions, cells, tissues, and organs [13–15]. This then reveals information about metabolic reactions and mechanisms that can help identify potential biomarkers of phenotypes of interest [14–16]. There have been some studies on the potential functions of metabolites in sperm physiology and their specific roles in metabolic pathways for reproductive success. For example, metabolic profiling analysis of mouse spermatozoa showed that the action of glycolytic substrates was associated with tyrosine phosphorylation and energy production, which is essential for flagella motility [17]. In the boar species, a study compared the pathways of glycolysis and gluconeogenesis to produce lactate/pyruvate and observed that the route of gluconeogenesis was limited use to produce energy for the spermatozoa [18]. Recently, using NMR and GC-MS techniques to determine metabolites in human spermatozoa, a total of 42 metabolites were revealed [19]. Also, supplementation of metabolites into extender medium used for liquid storage of goat sperm showed that glucose and pyruvate supplementation were better as compared to lactate for maintenance of sperm motility [20].

Metabolomics approaches have been also used to identify potential fertility biomarkers in the seminal plasma of bulls [11] and men [10, 20–26]. Moreover, a recent study reported that gas chromatography-mass spectrometry (GC-MS) can be an innovative method for fast and noninvasive diagnostic of man infertility [27]. Analyses of human seminal plasma by Raman spectroscopy allowed the identification of metabolites associated with asthenozoospermic patients as well [21]. In another study, GC-MS of seminal fluid detected lower concentrations of palmitic acid and oleic acid in healthy men as compared to asthenozoospermic individuals [26]. These findings suggest that metabolomic analyses are valid tools for identification of many classes of molecules associated with metabolic pathways essential to reproductive success [10, 12].

Although metabolomic tools have been used to study man’s fertility [20–27] there are only a limited number of such studies in farm animals. Vast gaps in the knowledge base exist, including metabolite identities, their concentrations in seminal plasma of bulls, as well as the molecular mechanism of their involvement in fertility. As such, the present study was conducted to perform a comprehensive analysis of the seminal plasma metabolome from adult Holstein bulls using GC-MS. In addition, we tested the hypothesis that differences exist in the seminal fluid metabolome between sires of contrasting *in vivo* fertility scores.

## Materials and methods

### Experimental design

Comprehensive metabolomics analysis of seminal plasma from Holstein bulls (n = 16) with contrasting *in vivo* fertility categories was performed using GC-MS. Following the analysis of metabolome data, computational biology tools were employed to detect potential biomarkers for bulls of high (n = 8) and low (n = 8) fertility.

### Sample collection and determination of bull fertility

Seminal plasma samples from 16 Holstein bulls with contrasting fertility phenotypes were provided by Alta Genetics (Watertown, WI, USA). All animals were raised under the same management conditions and received the same nutrition. Semen was collected with artificial vagina and seminal plasma was separated from sperm by centrifugation (700 × *g*, 4°C, 10 min). Supernatant seminal plasma was then transferred to a 2-mL microcentrifuge tube and centrifuged again (10,000 × *g*, 60 min, 4°C), as described previously [28]. After the second centrifugation, seminal plasma was aliquoted (100 μl) into a 2-mL Cryotube^®^ (Sigma-Aldrich, St Louis, USA), snap-frozen in liquid nitrogen, and transported to Mississippi State University (MSU). At MSU, seminal plasma was stored at -80°C until preparation for GC-MS.

In the present study, every ejaculate collected from each bull was routinely evaluated by standard semen analysis methods [29]. The method to determine bull fertility used in our study is also similar to previous investigations about fertility biomarkers in bulls, conducted by several authors in the last decades [4, 29–33]. The calculation of fertility scores was based on the actual conception rates confirmed by either veterinary palpation or ultrasound of cows inseminated with hundreds/thousands frozen-thawed semen straws from each bull. Sires used for this study were selected based on their fertility scores (Table 1), as previously described by Peddinti *et al*. [32]. Factors that influenced fertility performance of sires, e.g., breeding event, environmental factors and herd management were adjusted to determine reliable fertility scores using threshold models [34, 35]. Using Probit.F90 software [36], fertility prediction of each sire was calculated according to the average conception of more than 300 breeding outcomes along with their percent deviation of conception rates. Based on this calculation, percent deviation of conception rates was used to categorize the fertility of bulls.

**Table 1:** Fertility status of Holstein bulls.

Bull #	Bull number	Fertility status	Number of breedings	Conception rates % difference from average	Std of difference	Conception rates (%)
1	011HO10489	HF	5293	5.42	2.02	45.3
2	011HO11422	HF	825	5.1	1.90	40.4
3	011HO11436	HF	2032	4.8	1.79	40.3
4	011HO9748	HF	6378	4.6	1.67	44.4
5	011HO9212	HF	779	4.4	1.75	38.8
6	011HO11351	HF	2487	3.59	1.34	45.7
7	011HO11312	HF	5751	3.56	1.33	39.8
8	011HO9247	HF	1849	3.7	1.32	44.4
9	011HO11226	LF	1604	-3.75	-1.40	35.7
10	011HO11276	LF	2276	-4.06	-1.52	37.8
11	011HO11264	LF	967	-4.49	-1.68	34.4
12	011HO9354	LF	704	-5.6	-2.59	24.0
13	011HO9623	LF	747	-6.3	-2.91	26.7
14	011HO10928	LF	5603	-6.76	-2.52	34.6
15	011HO9415	LF	722	-7.5	-3.35	22.1
16	011HO11459	LF	674	-10.61	-3.96	23.3

Bulls 1 to 8 were defined as high fertility (HF) and bulls 9 to 16 were grouped as low fertility (LF). Fertility of each bull was expressed as the percent difference of its conception rate from the average conception rate of all bulls. Probit.F90 software was used to estimate fertility.

For this marker discovery study, bulls were selected among the ones performing conception rate of two standard deviation above and below the average of the bull population in the database. Thus, bulls which had percent difference of their conception rate above average were defined as high fertility (HF) with average of 3,174 breeding outcomes. Those bulls that had percent difference of their conception rate below average were classified as low fertility (LF) having 1,662 breeding outcomes (Table 1).

### Sample preparation for gas chromatography-mass spectrometry analysis

Preparation of seminal plasma samples for GC-MS analysis was performed as described by Shi *et al*. [37], with modifications. Briefly, a 100 μL aliquot of samples or reference standards (10 μM; Sigma-Aldrich, St Louis, USA) was thawed and pipetted into a 2-mL polypropylene microcentrifuge tube (Sigma-Aldrich, St Louis, USA). Then, 150 μL of heptadecanoic acid in methanol (1 mg/mL, internal standard; Sigma-Aldrich, St Louis, USA) and 350 μL of methanol were then added to the tube. This mixture was vortexed vigorously for 1 min and was then centrifuged at 12,000 × *g* at 4°C for 10 min. A volume of 100 μL of supernatant was transferred to a 2-mL amber glass vial (Agilent Technologies, Santa Clara, CA) and the solvent was evaporated to dryness in a TurboVap^®^ LV evaporator (Biotage, Charlotte, NC) with a gentle stream of nitrogen at 45°C.The dried extract was suspended in 50 μL of methoxyamine hydrochloride in pyridine (20 mg/mL; Sigma-Aldrich, St Louis, USA), vortexed vigorously for 1 min, and heated in a water bath at 70°C for 1 h. The sample was then derivatized by adding 100 μL of N,O-Bis(trimethylsilyl)trifluoroacetamide with 1% trimethylchlorosilane (BSTFA + 1% TMCS; Sigma-Aldrich, St Louis, USA) and heated again in a water bath at 70°C for 1 h. Derivatives of metabolites were transferred to an amber glass vial having a fixed insert (Agilent Technologies, Santa Clara, CA) for GC-MS analysis.

### Gas chromatography-mass spectrometry analysis

Samples and reference standards were analyzed using an Agilent 7890A GC System coupled to an Agilent 5975C inert XL MSD with triple-axis mass detector, an Agilent 7693 Series Autosampler, and a DB-5MS capillary column (30 m × 0.25 mm i.d. × 0.25 μm film thickness; Agilent Technologies, Santa Clara, CA). A volume of 1 μL of derivatized mixture was injected into the inlet heated at 280°C with 1:10 split ratio. Standard septum purge was performed after sample injection at 3 mL/min and helium carrier gas was at 1 mL/min constant flow rate. Transfer line, ion source, and quadrupole were heated at 250°C, 230°C, and 150°C, respectively. Oven was programmed initially at 70°C for 4 min, ramped up to 300ºC at 8°C/min, and then held at 300ºC for 5 min. Ionization was performed in an electron impact mode at 70 eV. Masses were scanned for full spectra from m/z 35 to 800 at 10,000 amu/s and 10.3 scans/s (m/z 0.2 step size). The solvent delay time was at 6 min.

### Calculation and statistical analysis

All identified metabolites were categorized according to their chemical classes. All compounds were identified by their retention times and one target and two quantitative ions in comparison with mass spectra of authentic standards and mass spectra in the NIST Mass Spectral Search Program (NIST/EPA/NIH Mass Spectral Library, Version 2.0). Abundances of the target ions of each metabolite were divided by that of target ion of the internal standard (heptadecanoic acid) and the unit less ratios were used for statistical analysis [37].

Multivariate analysis of abundance ratios was conducted by uploading data to MetaboAnalyst 3.0 (http://www.metaboanalyst.ca) [38]. The generated data matrix were normalized to a constant sum, auto-scaled, and analyzed by PLS-DA. VIP based on the PLS-DA model was calculated to identify the potential biomarkers and those variables with VIP score of more than 1.5 were considered important for group separation [11, 38, 39]. Univariate analyses were conducted using a simple t-test to evaluate the statistical significance between high fertility and low fertility bulls at *P* ≤ 0.05. Correlations among all seminal plasma metabolites were determined using Pearson's method (*P* ≤ 0.05) [38, 39]. In addition, Pearson's correlation (*P <* 0.05) was to determine the strength of the associations between seminal plasma metabolites (abundance ratios) and fertility scores (% deviation of conception rates).

### *In silico* analysis of metabolic networks

Bioinformatics tool was used to visualize metabolic networks and pathways. Metabolic networks related to the most abundant metabolites in bull seminal plasma, as well as for two metabolites with the highest VIP scores, were analyzed using Metscape version 3.1.2, which is a plug-in for Cytoscape version 3.2.1 (http://www.cytoscape.org). The Kyoto Encyclopedia of Genes and Genomes (KEGG) was used for compound identification [40].

## Results

### Metabolome of bull seminal plasma

**Fig 1:**
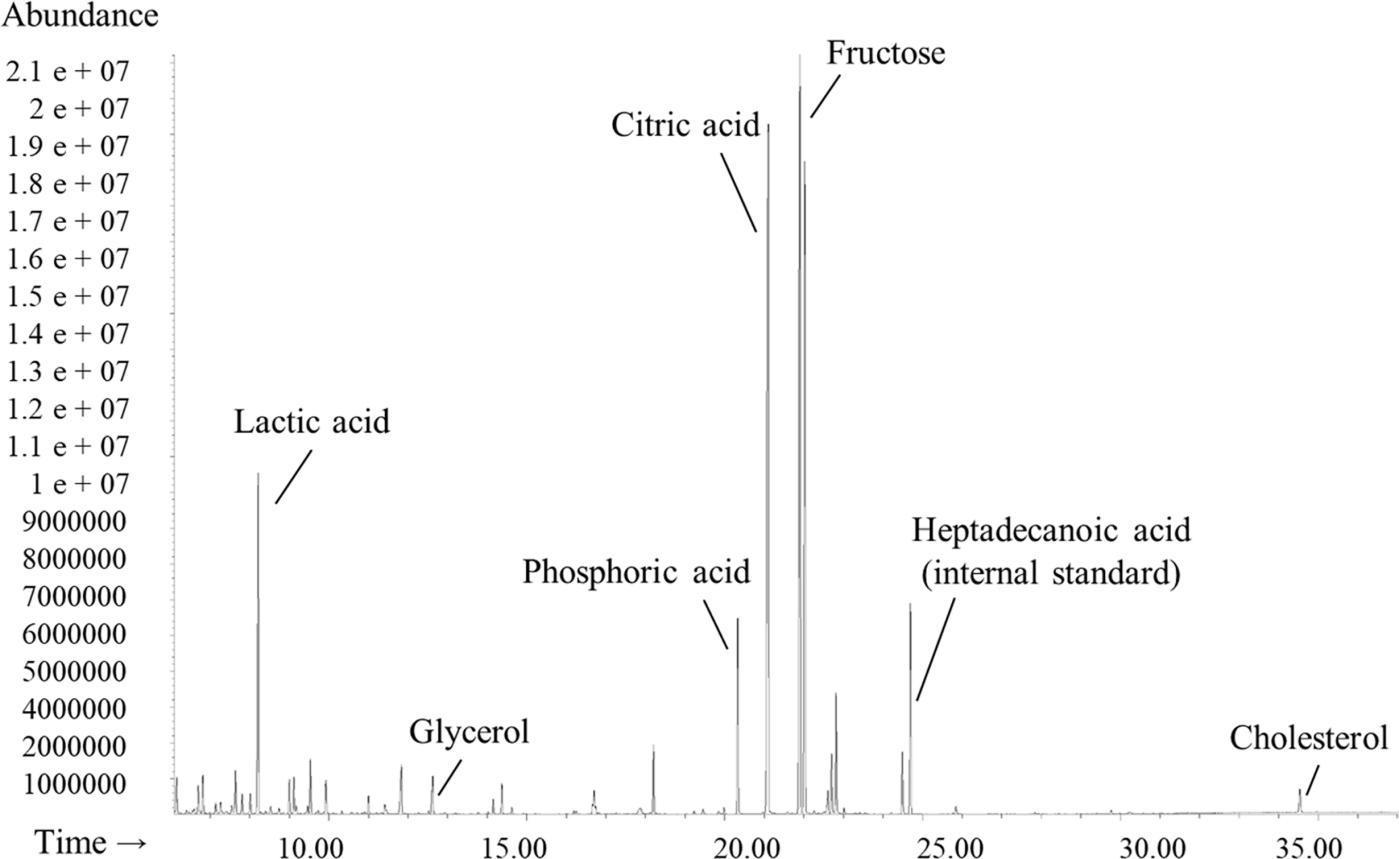
Representative GC-MS chromatogram of bull seminal plasma. Peaks of lactic acid, glycerol, phosphoric acid, citric acid, fructose, heptadecanoic acid (internal standard) and cholesterol are indicated.

A total of 73 peaks were integrated after GC-MS analysis of bull seminal plasma, regardless of fertility scores. Of these peaks, 63 metabolites were identified and categorized according to their major chemical classes, including amino acids, peptides/analogues, carbohydrates/carbohydrate conjugates, fatty acids/conjugates, steroids/steroid derivatives, nucleosides/nucleotides/analogues, and other organic and inorganic compounds (Table 2). A characteristic GC-MS chromatogram of bull seminal plasma and peaks of important metabolites were depicted in Fig 1. Of the 63 metabolites identified in the current study, 24 compounds were authenticated by external standard references and 39 compounds were identified by probable match parameters of the NIST Mass Spectral Search Program. In addition, retention time, target ion, and two quantitative ions, and chemical structure of the derivative product of each metabolite were also used for identification and quantification purposes (Table 2).

**Table 2:** Metabolites identified in bull seminal plasma by GC-MS.

**Metabolites and chemical class**	**Identified by**	**Retention time (minutes)**	**Target ion (m/z)**	**Quantitative ion (1) (m/z)**	**Quantitative ion (2) (m/z)**
***Amino acids, peptides, and analogues***					
**Amino-butyrolactone**	NIST	6.15	130	73	158
**L-alanine**	Standard	9.12	116	73	147
**Aminobutyric acid**	NIST	11.42	147	73	174
**Valine**	Standard	11.46	144	73	218
**L-leucine**	Standard	12.52	158	73	147
**L-isoleucine**	Standard	12.92	158	73	218
**Glycine**	Standard	13.13	130	73	147
**Norvaline**	Standard	13.58	232	73	144
**Serine**	Standard	14.15	204	73	218
**L-threonine**	Standard	14.62	218	73	117
**Beta-alanine**	Standard	15.20	174	73	147
**Aminomalonic acid**	NIST	15.96	147	73	218
**2-Pyrrolidone-5-carboxylic acid**	NIST	16.19	84	73	147
**Pyroglutamic acid**	Standard	16.70	156	73	147
**L-aspartic acid**	Standard	16.74	232	73	147
**L-proline**	Standard	16.83	140	73	230
**DL-ornithine**	Standard	18.12	142	174	420
**Glutamic acid**	Standard	18.20	246	73	128
**DL-phenylalanine**	Standard	18.27	218	73	192
**L-lysine**	Standard	22.27	174	73	317
**L-tyrosine**	Standard	22.48	218	73	147
***Carbohydrates and carbohydrate conjugates***					
**Glycerol**	Standard	12.63	147	73	205
**Meso-erythritol**	NIST	16.66	147	73	217
**Erythronic acid**	NIST	17.47	147	73	292
**D-xylofuranose**	NIST	17.51	82	73	110
**Ribitol**	NIST	19.89	103	73	147
**D-psicofuranose**	NIST	20.96	217	438	75
**D-fructopyranose**	NIST	21.16	204	73	147
**D-fructose**	Standard	21.90	103	73	217
**D-mannitol**	NIST	22.61	147	73	319
**D-sorbitol**	NIST	22.70	147	73	319
**D-glycero-D-gulo-heptose**	NIST	27.85	103	73	147
**Dulcitol**	NIST	27.93	103	73	147
***Fatty acids and conjugates***					
**Methylmaleic acid**	NIST	13.90	147	73	259
**Malic acid**	NIST	16.25	147	73	233
**Hexadecanoic acid**	Standard	23.55	117	73	132
**Stearic acid**	NIST	25.77	117	73	341
***Steroids and steroid derivatives***					
**Androstanedione**	NIST	29.01	147	73	91
**Cholesterol**	Standard	34.53	129	73	207
***Nucleosides*, *nucleotides*, *and analogues***					
**Inosine**	NIST	29.40	217	73	207
**5-Methyluridine**	NIST	29.77	217	73	147
***Others organic compounds***					
**Propylene glycol**	NIST	6.81	117	73	147
**Pyrrolidinone**	NIST	7.55	99	71	152
**Lactic acid**	NIST	8.21	117	73	147
**Pentane**	NIST	9.72	85	69	55
**Oxalic acid**	NIST	9.93	147	73	133
**Urea**	Standard	11.83	147	73	189
**Benzoic acid**	NIST	11.91	105	77	179
**Butanedioic acid**	NIST	13.20	147	73	247
**Fumaric acid**	NIST	13.78	245	73	147
**Creatinine enol**	NIST	17.26	115	73	100
**2-Oxoglutaric acid**	Standard	17.55	147	73	75
**Amino-methyl-propanediol**	NIST	17.89	188	73	100
**Aconitic acid**	NIST	19.98	229	67	375
**4-Ketoglucose**	NIST	20.05	89	59	392
**Hippuric acid**	NIST	20.72	105	73	206
**Citric acid**	Standard	21.10	147	73	273
**Carbonic acid**	NIST	22.82	71	58	147
**Myo-inositol**	NIST	24.49	147	73	217
**Uric acid**	NIST	24.53	442	73	457
**p-Tolyl-beta-D-glucuronide**	NIST	28.09	180	73	147
***Inorganic compounds***					
**Borate**	NIST	11.00	221	73	248
**Phosphoric acid**	NIST	20.33	299	73	357

Metabolites identified by external standard references and NIST library were classified based on their chemical class. Metabolites were identified by their retention time, one target ion, and two quantitative ions.

### Metabolites chemical classes

**Fig 2:**
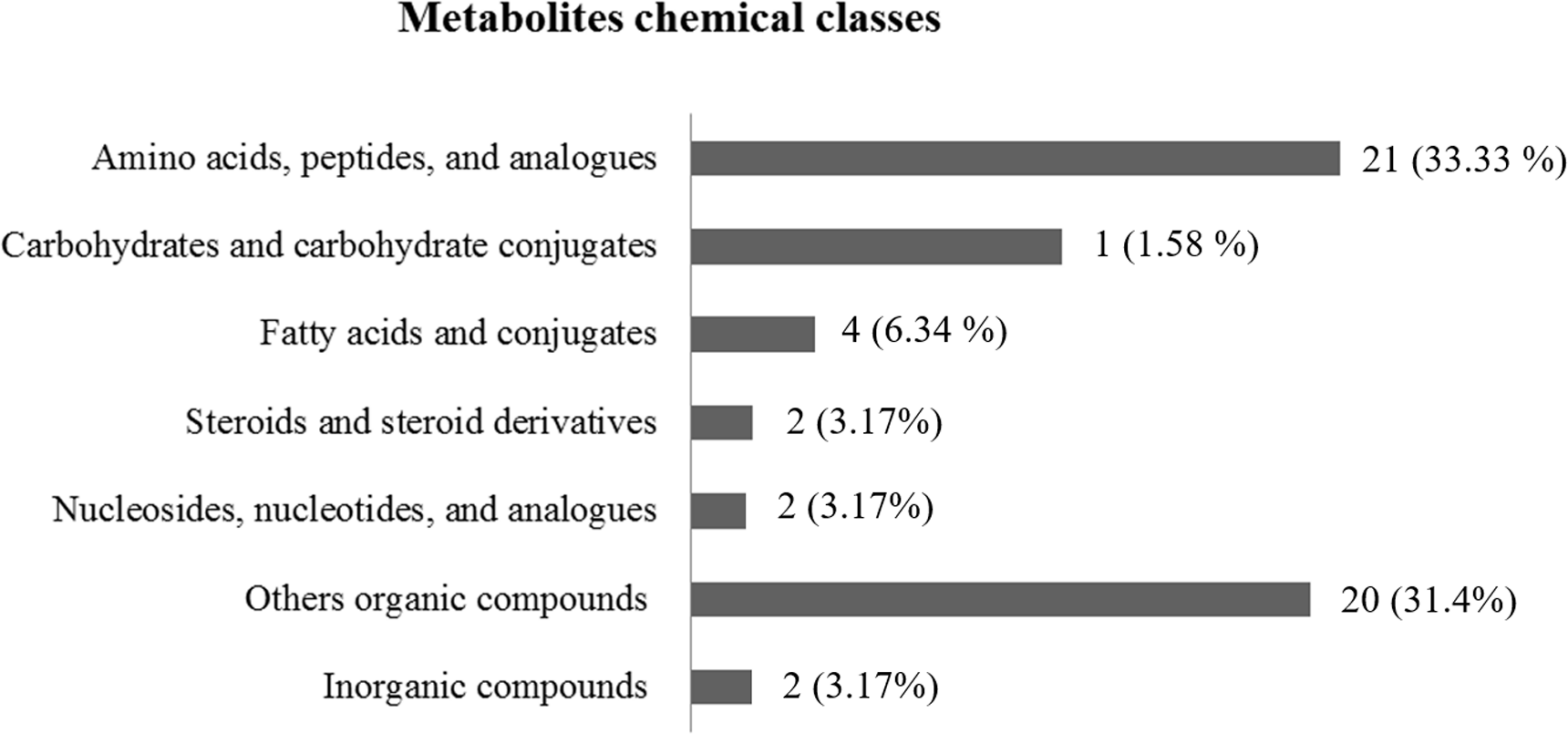
Number of metabolites per chemical class. Metabolites identified were categorized according to their chemical classes, defined as amino acids, peptides and analogues; carbohydrates and carbohydrate conjugates; fatty acids and conjugates; steroids and steroid derivatives; nucleosides, nucleotides, and analogues; others organic compounds and inorganic compounds.

Amino acids, peptides and analogues were the major compounds found in the bovine seminal plasma. Twenty-one amino acids were detected, including glutamic acid, alanine, isoleucine, leucine, and serine, among others. Twenty organic compounds, including citric acid, lactic acid, urea, uric acid, and myo-inositol comprised the second major group. Twelve carbohydrates and their conjugate metabolites were identified in the bull seminal plasma including fructose, mannitol, sorbitol, glycerol, and ribitol. In addition, we detected fatty acids and conjugates (malic acid, hexadecanoic acid, methylmaleic acid and stearic acid), steroids and steroid derivatives (androstenedione and cholesterol) and few nucleosides, nucleotides, and analogues (inosine and 5-methyluridine) and inorganic compounds (borate and phosphoric acid; Table 2 and Fig 2).

### Most and least predominant metabolites in the bull seminal plasma

**Fig 3:**
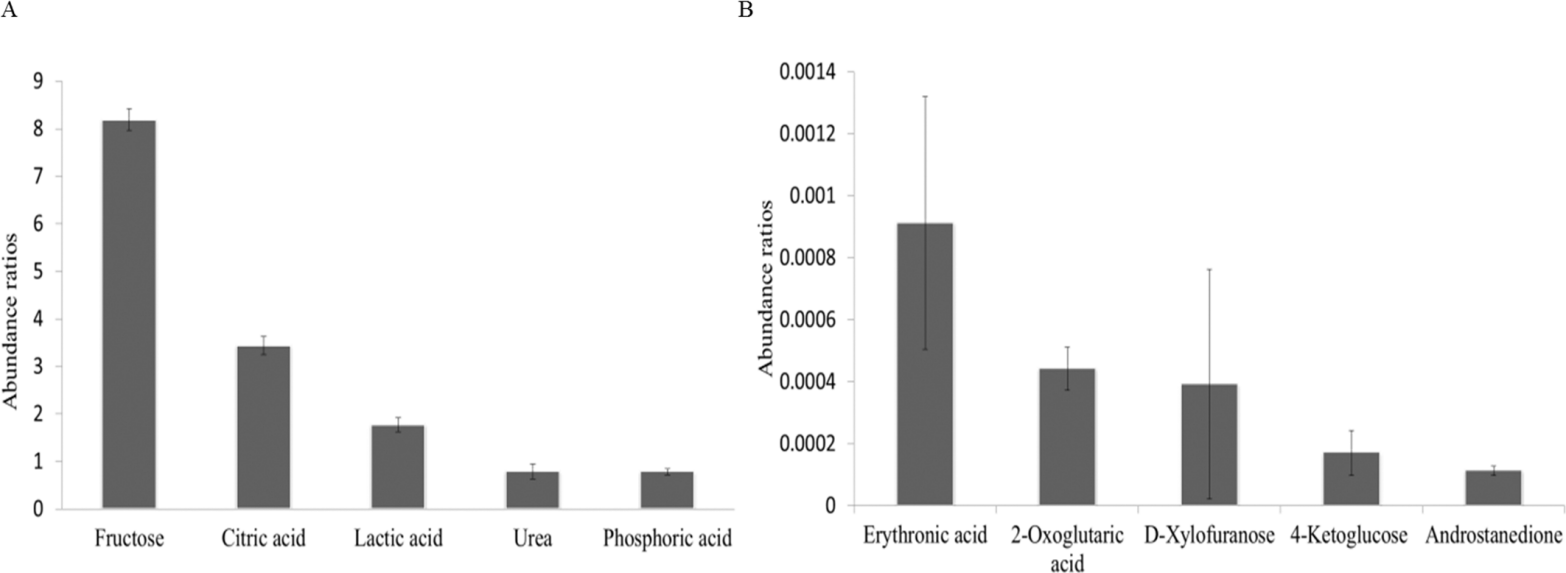
Abundance ratios of the most and least predominant metabolites present in bull seminal plasma. (A) The five most abundant metabolites were fructose, citric acid, lactic acid, urea and phosphoric acid and (B) the five least metabolites were identified as androstenedione, 4-ketoglucose, D-xylofuranose, 2-oxoglutaric acid and erythronic acid. Abundance ratio of the metabolites was calculated by dividing abundance of target ions of metabolites by that of target ion of the internal standard. Error bars represent standard error of the mean.

Fructose was the most predominant metabolite in seminal plasma of all bulls. Other predominant metabolites, based on their abundance ratios, were citric acid, lactic acid, urea and phosphoric acid (Fig 3A). Androstenedione, 4-ketoglucose, D-xylofuranose, 2-oxoglutaric acid and erythronic acid were among the five least predominant metabolites identified in bull seminal plasma (Fig 3B).

### Associations between seminal plasma metabolites and bull fertility

**Fig 4:**
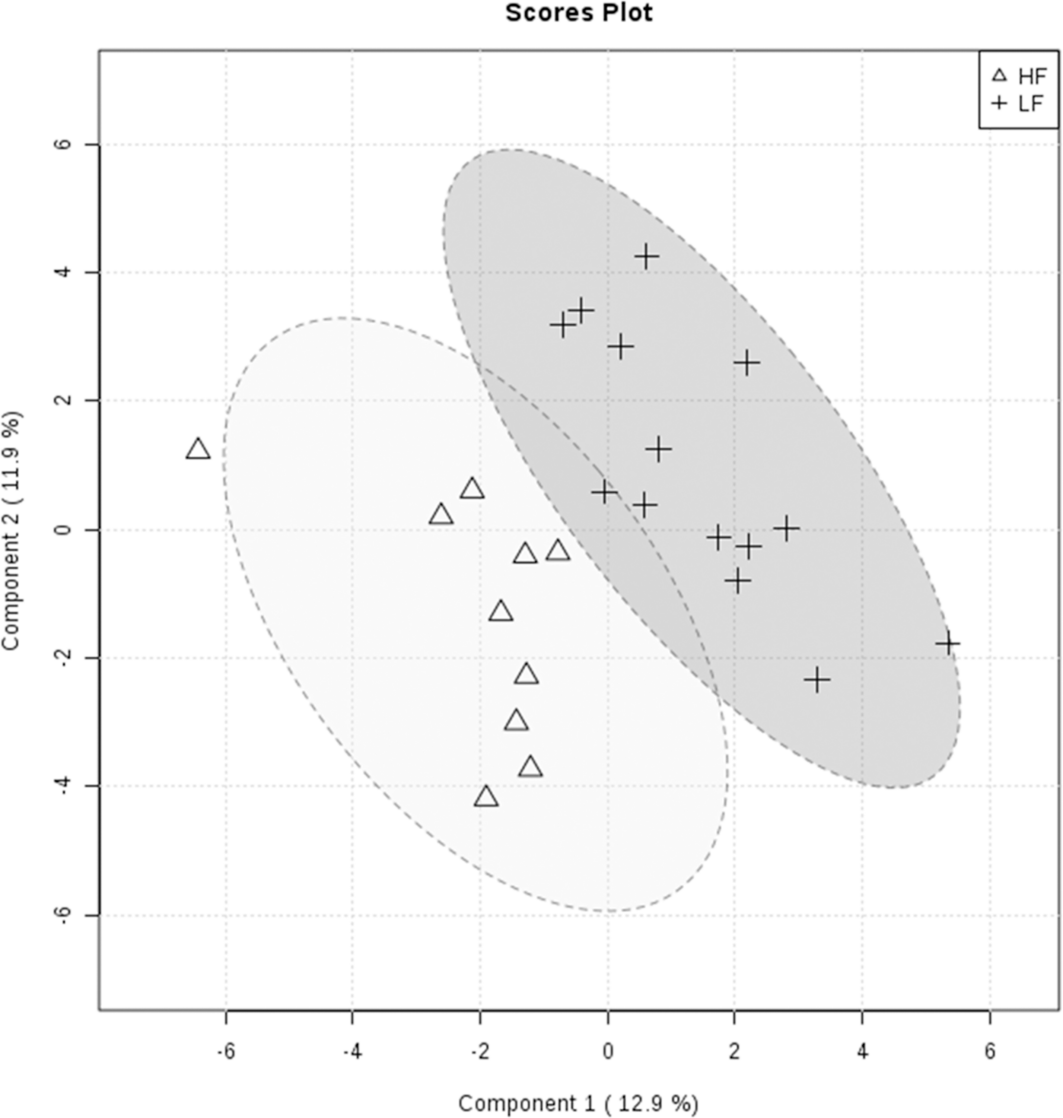
PLS-DA score plot of seminal plasma from high (HF) and low fertility (LF) bulls. The plots indicate that a separation could be observed between HF and LF bulls. Supervised PLS-DA was obtained with 2 components. The explained variances are shown in parentheses.

**Fig 5:**
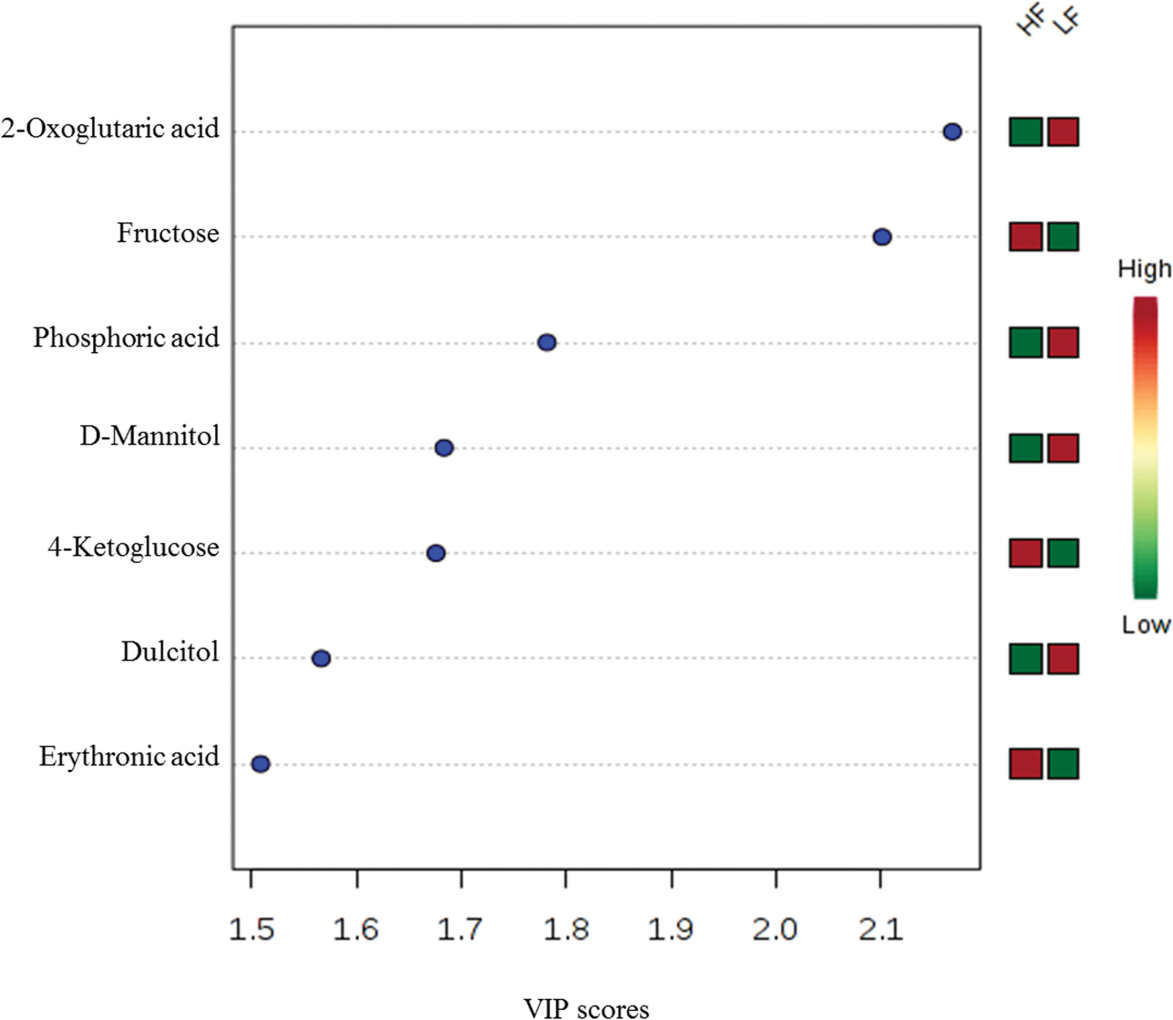
VIP scores of seminal plasma metabolites in high (HF) and low fertility (LF) bulls. The selected metabolites were those with VIP score of greater than 1.5. Heat map with red or green boxes on the right indicates high and low abundance ratio, respectively, of the corresponding metabolite in HF and LF bulls. VIP score was based on the PLS-DA model.

A multivariate analysis (Partial-Least Squares Discriminant Analysis; PLS-DA) of the seminal plasma metabolome indicated a distinct separation between high (HF) and low fertility (LF) bulls, as shown by PLS-DA score plot (Fig 4). In addition, metabolites with Variable Importance in Projection (VIP) score greater than 1.5 were identified as 2-oxoglutaric acid, fructose, phosphoric acid, D-mannitol, 4-ketoglucose, dulcitol and erythronic acid (Fig 5). Among these compounds, 2-oxoglutaric acid had the highest VIP score (VIP = 2.17), followed by fructose (VIP = 2.1). VIP score and the corresponding heat-map indicate the high or low abundance ratio of each metabolite in HF and LF bulls. Abundance ratios of 2-oxoglutaric acid, phosphoric acid, D-mannitol and dulcitol were lower in HF than in LF bulls. However, fructose, 4-ketoglucose and erythronic acid were more abundant in high fertility in comparison with low fertility bulls.

**Fig 6:**
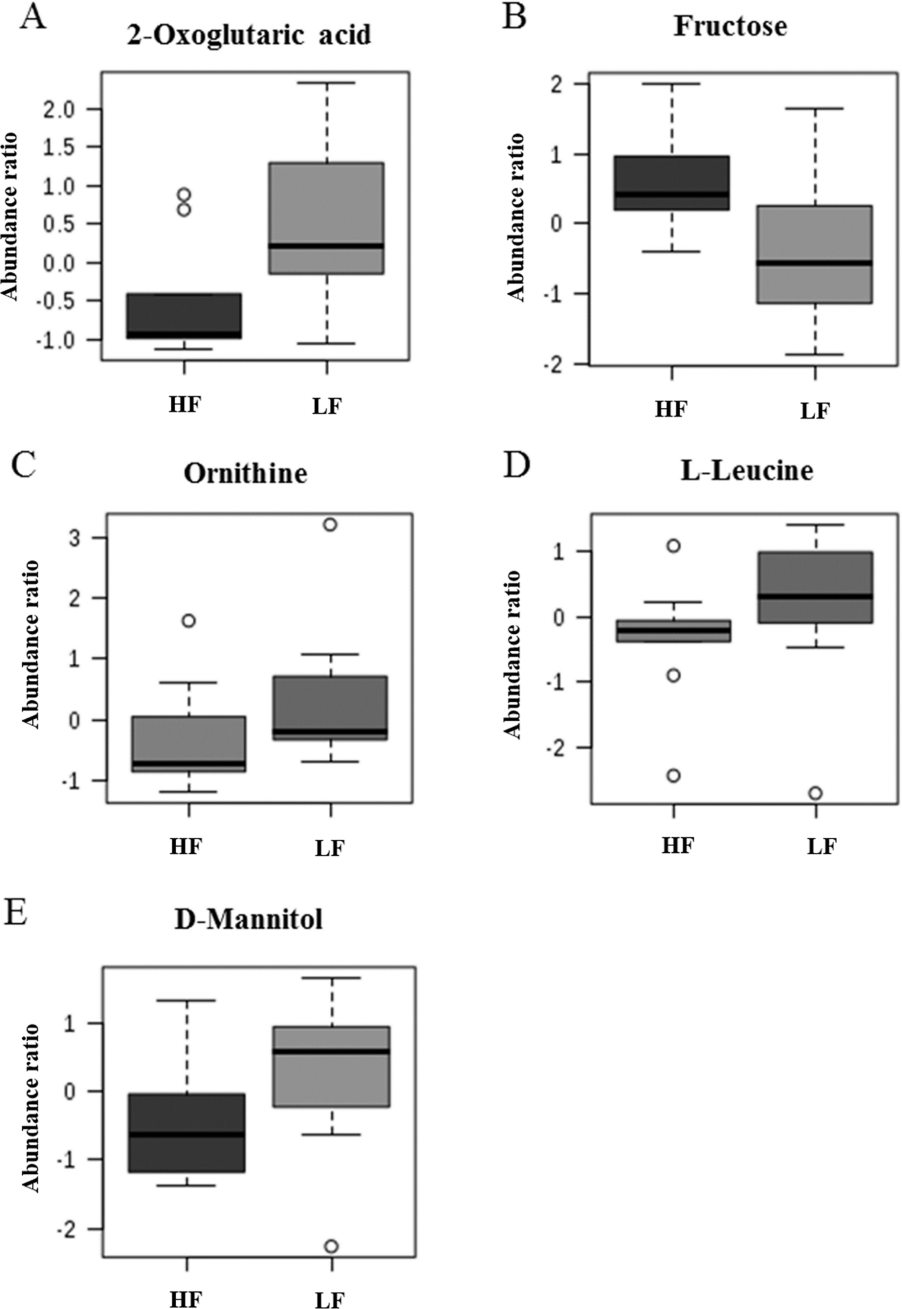
Box plots of the abundance ratio of five metabolites in high and low fertility bulls. (A) 2-oxoglutaric acid, (B) fructose, (C) ornithine, (D) L-leucine and (E) D-mannitol were significantly different (*P* ≤ 0.05) between high (HF) and low (LF) bulls.

**Fig 7:**
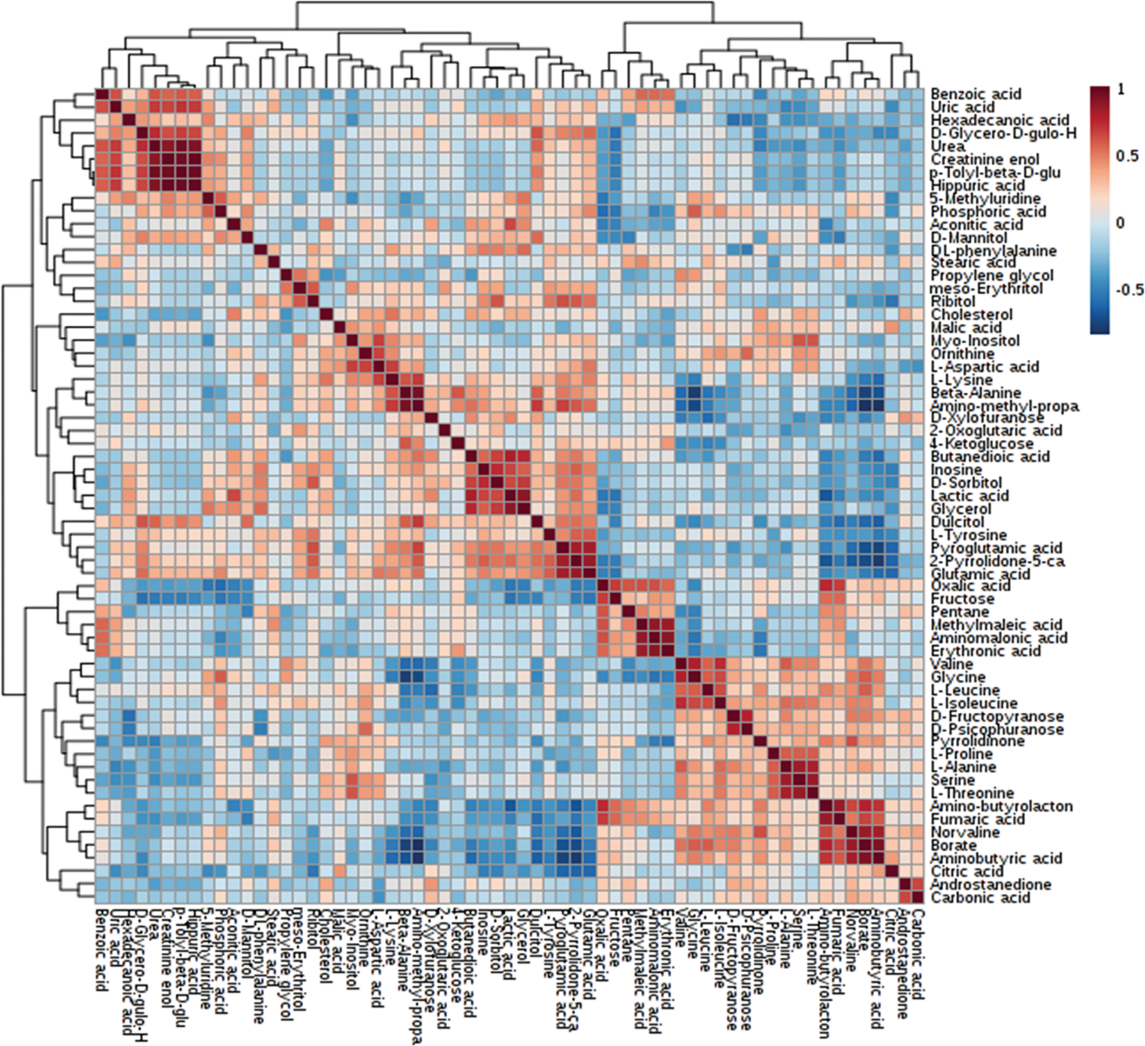
Heatmap of Pearson’s correlations among metabolites identified in bull seminal plasma.

Based on univariate analysis, abundance ratios of 2-oxoglutaric acid (*P* = 0.02), ornithine (*P* = 0.03), L-leucine (*P* = 0.04) and D-mannitol (*P* = 0.04) were lower in HF than in LF bulls, whereas abundance ratio of fructose was greater (*P* = 0.02) in HF as compared to LF bulls (Fig 6). Fructose was positively correlated with amino-butyrolactone (*r* = 0.55; *P* = 0.005), oxalic acid (*r* = 0.69; *P* < 0.0005) and fumaric acid (*r* = 0.57; *P* < 0.005), and inversely associated with p-tolyl-beta-D-glucuronide (*r* = -0.51; *P* = 0.01), lactic acid (*r* = -0.54; *P* = 0.006), urea (*r* = -0.52; *P* = 0.009), 2-pyrrolidone-5-carboxylic acid (*r* = -0.51; *P* = 0.01), glutamic acid (*r* = -0.53; *P* = 0.008), phosphoric acid (*r* = - 0.54; *P* = 0.007) and D-glycero-D-gulo-heptose (*r* = -0.56; *P* = 0.004) (Fig 7).

Based on Pearson’s method, abundance ratio of ornithine (r = 0.59; P = 0.02) was significantly associated with fertility score of LF bulls. In addition, there was also a positive and significant correlation between abundance ratio of D-mannitol (r = 0.53; P = 0.04) and fertility score of LF bulls.

### Metabolic networks of seminal plasma metabolites

**Fig 8:**
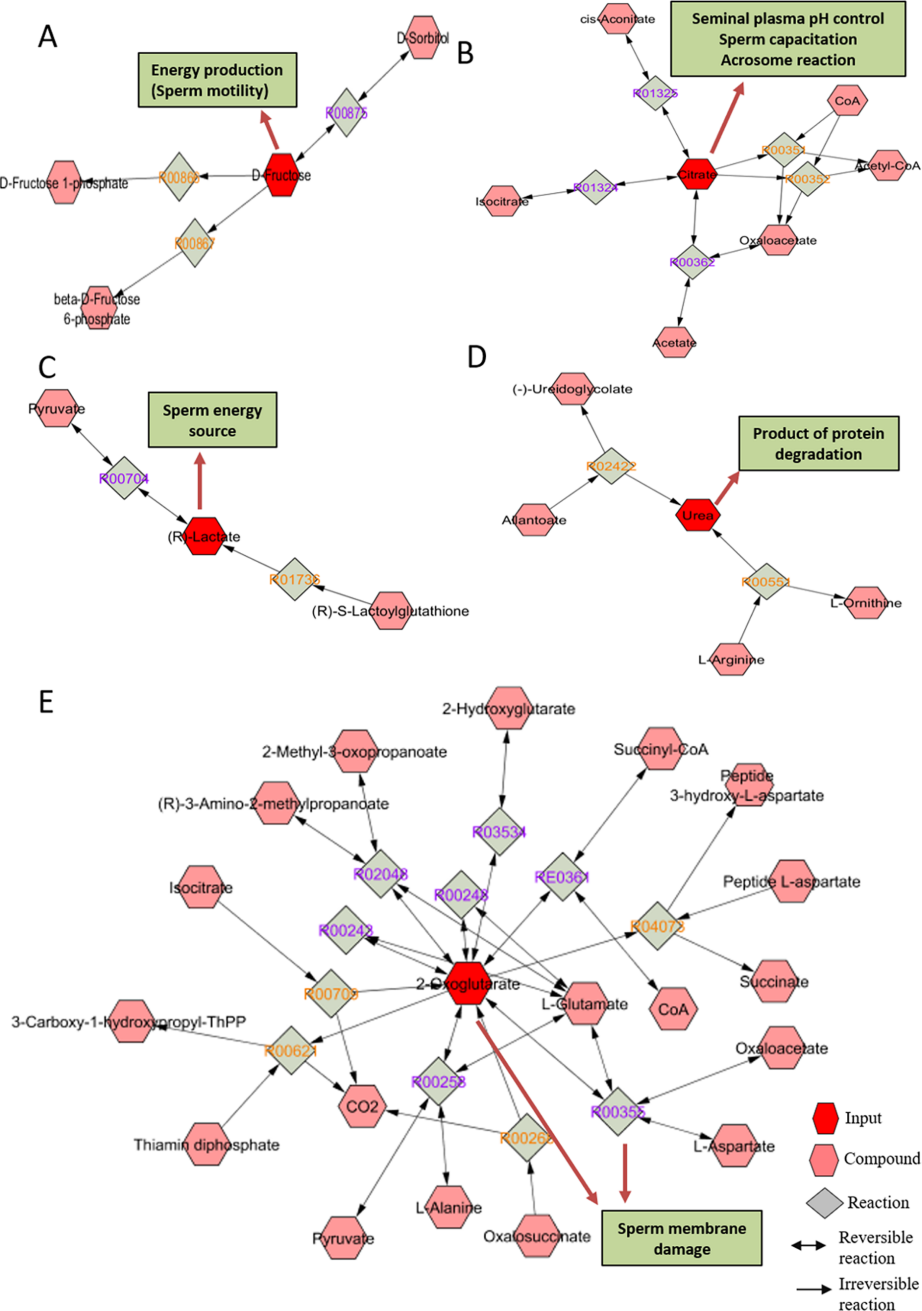
Metabolic networks associated with metabolites identified in bull seminal plasma. (A) fructose, (B) citric acid, (C) lactic acid, (D) urea, (E) 2-oxoglutaric acid. Networks were generated using the Metscape plug-in for Cytoscape. Metabolites are shown in red hexagons. Compounds are represented as pink hexagons, reversible reactions as gray squares with purple text and non-reversible reactions as gray squares with orange text.

Metabolic networks were determined for fructose, citric acid, lactic acid, and urea (the most abundant seminal plasma metabolites) as well as for 2-oxoglutaric acid (metabolite with the highest VIP score). Based on fructose network panel (Fig 8A), D-sorbitol undergoes a reversible reaction to produce fructose, which is converted to beta-D-fructose-6-phosphate and D-fructose-1-phosphate. Citric acid can be interconverted to cis-aconitate, isocitrate, acetate and oxaloacetate. Then, non-reversible reactions yield acetyl-CoA and oxaloacetate (Fig 8B). Lactic acid is synthesized from S-lactoylglutathione or pyruvate in a non-reversible and reversible reaction, respectively (Fig 8C). Urea can be produced from L-arginine and allantoate, which is converted to urea and ureidoglycolate. L-arginine is hydrolyzed to urea and ornithine (Fig 8D). Furthermore, 2-oxoglutaric acid is involved in several reversible and non-reversible reactions, as it can be synthetized from L-glutamate, succinyl-CoA, 2-hydroxyglutarate, 2-methyl-3-oxopropanoate and 3-amino-2-methylpropanoate, among others. It can be converted to 3-carboxy-1-hydroxypropyl-ThPP, succinate and peptide-3-hidroxy-L-aspartate (Fig 8E). Reactions and enzymes of each metabolite network are presented in S1 Table.

## Discussion

In the present study, we performed GC-MS analysis to determine the metabolite profile of bull seminal plasma and identify potential biomarkers of fertility. Additionally, we used bioinformatics tools to reveal the networks and reactions in which bull seminal plasma metabolites might be involved. A previous study has investigated amino acid and fatty acid composition of bovine seminal plasma using GC-MS [41]. To the best of our knowledge, however, our study is in fact the first to conduct a comprehensive evaluation of the bull seminal plasma metabolome, which includes not only amino acids and fatty acids but also carbohydrates, nucleosides, steroids, organic and inorganic compounds by GC-MS. Moreover, we described the association of specific seminal plasma metabolites with bull fertility scores measured *in vivo*.

Metabolites, products of metabolic reactions, appear in numerous biochemical pathways [42] and have been reported as potential biomarkers for male fertility [10, 20, 25–27]. At ejaculation, spermatozoa are suspended in the seminal plasma and it marks clear qualitative and quantitative differences in its biochemical composition [43]. Although the effects of the seminal plasma are still not clearly understood, the exposure of spermatozoa to small molecules such as metabolites can improve or hinder sperm fertilizing capacity, even if semen is diluted in extender during preservation of spermatozoa. The removal of seminal plasma is not necessary for bull sperm preservation and the timeline of the preservation protocol (e.g., a long cooling curve and equilibrium step) leads to a prolonged exposure of spermatozoa to seminal plasma molecules [44]. In addition, the interaction of metabolites with other molecules in the uterine environment impacts fertilization, implantation, and optimal fetal and placental developments [5]. Furthermore, metabolites such as amino acids, peptides, carbohydrates, fatty acids, steroids and nucleosides, among others, participate in physiologically important events, influencing energy production, motility, pH control, membrane protection and metabolic activity of the sperm [22, 45–47].

As presently evaluated by GC-MS, the major chemical classes of metabolites in the bovine seminal plasma were defined as amino acids, peptides, and their analogues, followed by carbohydrates and carbohydrate conjugates. We identified 21 metabolites classified as amino acids, peptides, and their analogues in bull seminal plasma. Similarly, researchers have detected 20 [48] to 23 amino acids [41] in seminal plasma of bulls, using GC-MS. A large number of amino acids were also found in human sperm [19] and in goat epididymal fluid [47]. Besides playing essential roles as basic building block of proteins [49], amino acids participate in crucial steps of sperm biology, including protection and regulation of metabolic activity [47]. Amino acids protect ram sperm cells during cryopreservation by decreasing lipid peroxidation and injury caused by free-radicals [50]. Meanwhile, the presence of carbohydrates in mammalian seminal plasma is essential for sperm because these molecules are part of crucial energy production pathways [46], and glycolysis is used by mammalian sperm to obtain energy. During this event, seminal plasma glycolysable carbohydrates, such as fructose, are required for ATP production, which leads to increased respiratory activity to support optimum sperm motility and survival [51, 52].

The most predominant metabolites of the bull seminal plasma were fructose, citric acid, lactic acid, urea and phosphoric acid; whereas androstenedione, 4-ketoglucose, D-xylofuranose, 2-oxoglutaric acid and erythronic acid were among the least abundant. Multivariate statistical analysis showed that fructose abundance separated HF from LF groups and it had the second highest VIP score. Fructose abundance ratio was higher (*P* = 0.02) in HF than in LF bulls. Fructose is the primary energy source for bull spermatozoa and the major carbohydrate in seminal plasma of these animals [52–54]. Fructose is produced in the seminal vesicles, under androgen stimulation [46], and it has been found in seminal plasma of several species, including buffalo [55], goat [55, 56], ram [57], boar [58], human [59], and rabbit [60]. As revealed by *in silico* network analysis, fructose is involved in fundamental pathways for energy production for the spermatozoa. Fructose can be produced from sorbitol by the action of sorbitol dehydrogenase, which is located on the sperm membrane. Once fructose enters the cell, it is converted to beta-D-fructose-6-phosphate and fructose-1-phosphate [53, 61]. Furthermore, fructose can be metabolized to lactic acid, depending on various factors such as pH and temperature [52, 53]. Smaller fructose concentration has been found in the seminal plasma of azoopermic, oligozoospermic, and idiopathic infertile man compared to fertile man [59]. Yousef et al. [60] suggested that a decrease in fructose concentration observed in the seminal plasma of rabbits intoxicated with aluminum chloride could be one of the factors reducing sperm motility. Therefore, less fructose abundance in bull seminal plasma reduces energy supply to sperms, negatively affecting their metabolism and, subsequently, male fertility.

As in bull seminal plasma, citric acid is also found in semen of other species, such as boar [62], human [26, 59], and rabbit [63]. Citric acid helps control pH in boar semen and acts as a chelator for zinc, magnesium, and calcium [62]. The concentration of zinc, magnesium and calcium in human seminal plasma and their chelation can influence sperm metabolism, affecting sperm transport, acrosome reaction, and fertilization [64]. A recent study demonstrated that citric acid in seminal plasma is associated with bull fertility by potentially affecting sperm capacitation and acrosome reaction [11]. Lactic acid, like fructose and citric acid, is used as another important energy source for boar sperm [65] and lesser concentration of lactic acid has been detected in bull sperm with low viability [66]. Both urea and phosphoric acid were detected in the seminal plasma samples of our study. Urea has also been found in human seminal fluid [20, 26, 67], however, the main role it plays in semen is still unknown. Given that urea is an end product of protein metabolism [68] and that seminal fluid contains protein, it is possible that seminal plasma urea is the consequence of protein degradation. We also observed that phosphoric acid had the third highest VIP score, meaning that this metabolite is associated with lower fertility scores of the Holstein bulls. In spermatozoa, phosphoric acid can be a product of a reaction catalyzed by inorganic pyrophosphatase (PPA1). PPA1 catalyzes the hydrolysis of one molecule of inorganic pyrophosphate (PPi) to two molecules of phosphoric acid, leading to the release of energy. The transport of PPi from spermatozoa to the seminal plasma may be regulated by a transmembrane protein, called progressive ankylosis protein (ANKH). Therefore, the energy produced from the conversion of PPi to phosphoric acid could be utilized for sperm motility and during fertilization [69]. Moreover, inorganic phosphate can be resulted from the hydrolysis of ATP used for metabolism and sperm motility. Presence of greater abundance of inorganic phosphate indicated an increase in level of either motility or metabolism during ejaculation. At this point, it is unclear why greater inorganic phosphate was associated with LF group. We hypothesized that increased activity during ejaculation led to decreased reservation of energy, thereby decreasing fertility.

In the current study, propylene glycol was identified in bull seminal plasma. This finding is in agreement with previous studies showing that propylene glycol is present in human sperm [19], serum [70] and urine [71]. In addition, propylene glycol, a synthetic molecule, is found in pharmaceutical products and in food that are routinely used for animal feeding. As a matter of fact, propylene glycol has been used as feed additive for dairy cattle [72]. Therefore, we believe that the presence of propylene glycol in bull seminal plasma is not the result of contamination since this molecule has also been detected in human cells and secretions. However, it is possible that propylene glycol originally comes from feeds and it may also be the reason it was found in human and animal samples. The role of this compound in sperm fertility is currently unknown.

The compound identified as 2-oxoglutaric acid was one of the least abundant metabolites in bull seminal plasma but had the greatest VIP score, being less abundant in high fertility than in low fertility bulls. In agreement with our results, 2-oxoglutaric acid was found in greater concentration in the seminal plasma of men with asthenozoospermia than in that of the healthy ones [73]. It is known that 2-oxoglutaric acid can be synthesized from glutamate by 2-oxoglutarate aminotransferase [74], as also observed by the in silico analysis conducted in the present study. In boar semen, 2-oxoglutarate aminotransferase was mainly detected in the spermatozoa [75] and 2-oxoglutarate aminotransferase was released at high levels from boar sperm with low viability after semen freezing [76]. Other experiments proposed that high activity of 2-oxoglutarate aminotransferase in seminal plasma is an indicator of sperm membrane injury and poor semen quality in goats and bulls [77–79]. Therefore, all these findings agree with our work reporting the occurrence of higher abundance ratio of 2-oxoglutaric acid content in seminal plasma of low fertility bulls.

In recent decades, *omics* approaches have been used to identify several seminal plasma macromolecules associated with male fertility [29, 30, 80, 81]. Using 2-D gels and mass spectrometry, an interesting study on the investigation of capacitation-related proteins in boar spermatozoa identified, after *in vitro* capacitation, a significant increase in tyrosine-phosphorylated proteins. The results of this study revealed differentially expressed proteins involved in cellular processes and biological regulation. This finding is important not only as related to semen physiology, but also to determine *in vivo* fertility in the boar species [82]. Another proteomic work was conducted to evaluate differences in protein profiles between high- and low-litter sizes in boar spermatozoa in a field trial [83]. The study uncovered proteins potentially involved in the regulation of male fertility by contributing in capacitation, acrosome reaction, sperm-egg interaction, and fertilization processes. These results provide insights into the role played by these proteins in sperm physiology and male fertility. Fertility-related proteins in capacitated boar spermatozoa were also reported by Kwon *et al*. [84]. The study showed proteins differentially expressed between high- and low-litter size spermatozoa (> 3-folds) involved in sperm physiology. In addition, ras-related protein Rab-2A and cytochrome b-c1 complex subunit 1 expression negatively associated with litter size spermatozoa whereas cytochrome b-c1 complex subunit 2 was positively correlated with litter size spermatozoa. These results suggest that those proteins found in sperm following capacitation could be helpful as indicators of male fertility [84]. Proteins present in seminal plasma along with spermatozoa may contribute to improvement of fertilizing capacity of the sperm. In boar seminal plasma, fibronectin-1, one of the most abundant proteins in boar seminal plasma, was identified [85]. The presence of fibronectin-1 and its association with sperm function demonstrates that seminal plasma has potentially relevant biomarkers for cryoinjury [86, 87]. Using bull epididymal spermatozoa as a model, a comprehensive proteomic study employed 2D electrophoresis technique and identified proteins associated with cryostress and their associated signaling pathways, including the ephrinR-actin pathway, the ROS metabolism pathway, actin cytoskeleton assembly, actin cytoskeleton regulation, and the guanylate cyclase pathway [88]. Another study showed evidences that the addition of cryoprotectant alters the bull epididymal sperm proteome [89]. The authors also observed that NADH dehydrogenase flavoprotein 2, f-actin-capping protein subunit beta, superoxide dismutase 2, and outer dense fiber protein 2 were associated with several important signaling pathways. These findings are useful to understand the mechanisms of cryoprotectant in protecting bull spermatozoa and for the development of novel extenders. Metabolomics technology emerged as a means of understanding physiological and pathological conditions in a large-scale manner, through the identification of metabolic substrates and products of a given biochemical system. Considering the metabolome as the metabolic state of a given physiologic status of a given fluid, cell, tissue, or organism, metabolomics is not only a complementary tool for understanding proteomics data, but also to gain insight into biochemical reaction networks, to understand mechanistically how the metabolites may affect male fertility as well as potential biomarker discovery.

In conclusion, we have demonstrated that fructose, citric acid, lactic acid, urea and phosphoric acid are the predominant metabolites in bull seminal plasma. We discovered a clear separation of metabolite profiles between high and low fertility bulls, with fructose and 2-oxoglutaric acid being potential candidates for biomarkers of bull fertility. Findings of the present study will help advance our current understanding of the multifactorial and complex processes related to the physiology of male fertility.

## Supporting information

S1 Table Reactions and enzymes of metabolite networks identified in bull seminal plasma. (DOCX)
