# Supplementary material for: Metabolomic markers of fertility in bull seminal plasma
Source: PLoS One. 2018 Apr 10;13(4):e0195279. doi: 10.1371/journal.pone.0195279 (PMC5892889; doi:10.1371/journal.pone.0195279)
Supplement: S1 Table — (DOCX) [file pone.0195279.s001.docx]

# S1 Table. Reactions and enzymes of metabolite networks identified in bull seminal plasma

| **Metabolite network** | **KEGG reaction** | **Reaction** | **Enzyme** |
| --- | --- | --- | --- |
| Fructose  (A) | R00866 | ATP + D-Fructose <=> ADP + D-Fructose 1-phosphate | ATP:D-fructose 1-phosphotransferase |
|  | R00867 | ATP + D-Fructose <=> ADP + beta-D-Fructose 6-phosphate | ATP:D-fructose 6-phosphotransferase |
|  | R00875 | D-Sorbitol + NAD+ <=> D-Fructose + NADH + H+ | D-Glucitol:NAD+ 2-oxidoreductase |
| Citrate  (B) | R01325 | Citrate <=> cis-Aconitate + H2O | citrate hydro-lyase (cis-aconitate-forming) |
|  | R01324 | Citrate <=> Isocitrate | citrate hydroxymutase |
|  | R00362 | Citrate <=> Acetate + Oxaloacetate | citrate oxaloacetate-lyase (forming acetate from the pro-S carboxymethyl group of citrate) |
|  | R00351 | Citrate + CoA <=> Acetyl-CoA + H2O + Oxaloacetate | acetyl-CoA:oxaloacetate C-acetyltransferase (thioester-hydrolysing) |
|  | R00352 | ATP + Citrate + CoA <=> ADP + Orthophosphate + Acetyl-CoA + Oxaloacetate | acetyl-CoA:oxaloacetate C-acetyltransferase [(pro-S)-carboxymethyl-forming, ADP-phosphorylating] |
| Lactate  (C) | R00704 | (R)-Lactate + NAD+ <=> Pyruvate + NADH + H+ | (R)-Lactate:NAD+ oxidoreductase |
|  | R01736 | (R)-S-Lactoylglutathione + H2O <=> Glutathione + (R)-Lactate | (R)-S-Lactoylglutathione hydrolase |
| Urea  (D) | R00551 | L-Arginine + H2O <=> L-Ornithine + Urea | L-Arginine amidinohydrolase |
|  | R02422 | Allantoate + H2O <=> (S)-Ureidoglycolate + Urea | Allantoate amidinohydrolase |
| 2-Oxoglutaric acid  (E) | R00243 | L-Glutamate + NAD+ + H2O <=> 2-Oxoglutarate + Ammonia + NADH + H+ | L-glutamate:NAD+ oxidoreductase (deaminating) |
|  | R00248 | L-Glutamate + NADP+ + H2O <=> 2-Oxoglutarate + Ammonia + NADPH + H+ | L-Glutamate:NADP+ oxidoreductase (deaminating) |
|  | R03534 | 2-Hydroxyglutarate + FAD <=> 2-Oxoglutarate + FADH2 | 2-Hydroxyglutarate:(acceptor) 2-oxidoreductase |
|  | R00709 | Isocitrate + NAD+ <=> 2-Oxoglutarate + CO2 + NADH + H+ | isocitrate:NAD+ oxidoreductase (decarboxylating) |
|  | R04073 | Peptide L-aspartate + 2-Oxoglutarate + Oxygen <=> Peptide 3-hydroxy-L-aspartate + Succinate + CO2 | Peptide-L-aspartate,2-oxoglutarate:oxygen oxidoreductase (3-hydroxylating) |
|  | R00621 | 2-Oxoglutarate + Thiamin diphosphate <=> 3-Carboxy-1-hydroxypropyl-ThPP + CO2 | 2-ketoglutarate dehydrogenase |
|  | R00258 | L-Alanine + 2-Oxoglutarate <=> Pyruvate + L-Glutamate | L-Alanine:2-oxoglutarate aminotransferase |
|  | R00268 | Oxalosuccinate <=> 2-Oxoglutarate + CO2 | oxalosuccinate carboxy-lyase (2-oxoglutarate-forming) |
|  | R00355 | L-Aspartate + 2-Oxoglutarate <=> Oxaloacetate + L-Glutamate | L-Aspartate:2-oxoglutarate aminotransferase |
